# Maternal Vitamin D Status, Gestational Hypertension, and Preeclampsia: A Cross-Sectional Study in Urban Greece

**DOI:** 10.3390/biomedicines13112624

**Published:** 2025-10-27

**Authors:** Artemisia Kokkinari, Kleanthi Gourounti, Maria Dagla, Nikoleta Tsinisizeli, Georgios Iatrakis

**Affiliations:** 1 Department of Midwifery, School of Health & Care Sciences, University of West Attica, 12243 Athens, Greece; kgourounti@uniwa.gr (K.G.); mariadagla@uniwa.gr (M.D.); nikoletatsinisizeli@gmail.com (N.T.); giatrakis@uniwa.gr (G.I.); 2 General Hospital of Nikaia “Agios Panteleimon”, 18454 Nikea, Greece

**Keywords:** maternal vitamin D, neonatal vitamin D, gestational hypertension, preeclampsia, hypovitaminosis D, birth outcomes, Mediterranean population

## Abstract

**Background**: Evidence linking maternal vitamin D status with gestational hypertensive disorders and neonatal outcomes in Southern Europe remains limited. We evaluated maternal and cord 25-hydroxyvitamin D [25(OH)D] at birth in an urban Greek cohort and examined associations with gestational hypertension and preeclampsia. **Methods**: We conducted a cross-sectional study of 248 mother–infant dyads delivering at Tzaneio General Hospital of Piraeus, Greece. Eligible participants were of Greek origin or long-term residents (>10 years). Maternal venous and umbilical cord blood were obtained at birth and analyzed for serum 25(OH)D. Postpartum questionnaires captured sun exposure, supplement use, and selected lifestyle factors; clinical and obstetric data, including diagnoses of gestational hypertension and preeclampsia, were abstracted from medical records. We classified 25(OH)D as deficient (<20 ng/mL), insufficient (20–29 ng/mL), and, for risk-stratified analyses, treated values < 30 ng/mL as low. **Results**: Maternal 25(OH)D concentrations varied seasonally (winter 16.96 ± 9.60 ng/mL; summer 24.22 ± 12.57 ng/mL) and correlated with cord concentrations (r = 0.80). Most mothers (75–89%) had <30 ng/mL across seasons, and 73% of neonates were <20 ng/mL despite supplementation. Gestational hypertension occurred in 29/248 (11.7%) and preeclampsia in 15/248 (6.0%), with low maternal 25(OH)D common among affected women. **Conclusions**: In this cross-sectional study of an urban Mediterranean population, hypovitaminosis D was highly prevalent among mothers and neonates, with seasonal variation and clustering among hypertensive pregnancies. These findings support prenatal care strategies beyond fixed supplementation, incorporating season- and environment-sensitive dosing with screening and dietary counseling. Prospective studies are needed to clarify causality and refine supplementation targets.

## 1. Introduction

Hypertensive disorders of pregnancy (HDP), encompassing gestational hypertension and preeclampsia, affect approximately 5–10% of pregnancies worldwide and remain among the leading causes of maternal and perinatal morbidity and mortality [1,2]. Given this global burden, identifying modifiable determinants such as vitamin D status has gained increasing scientific attention. Despite substantial progress in obstetric care, the multifactorial pathophysiology of these disorders is not yet fully elucidated, and the identification of modifiable risk factors continues to be a major research priority [3]. Emerging evidence suggests that vitamin D deficiency (VDD) may represent one such factor. Vitamin D plays a central role not only in calcium–phosphate metabolism but also in immune regulation, endothelial function, and placental development, mechanisms that may influence the risk of HDP [3,4]. A recent umbrella review of systematic reviews and meta-analyses by Chien et al. [5] synthesized data from over 40 studies and confirmed a possible link between low maternal 25(OH)D concentrations and an increased risk of hypertensive disorders of pregnancy, although findings remain heterogeneous and causality unproven [5].

The investigation aims to quantify the prevalence of vitamin D insufficiency among mothers and their neonates at term, to delineate associations between maternal vitamin D levels and the development of hypertensive complications, and to evaluate whether vitamin D deficiency (VDD) acts as an independent risk factor when accounting for relevant confounders such as body mass index (BMI), socioeconomic status, supplementation, and parity. By incorporating data on seasonal variations, supplementation habits, and maternal–neonatal biomarkers, the study addresses a critical knowledge gap in Mediterranean populations, where abundant sunlight does not necessarily guarantee adequate vitamin D status [6], with prevalence often reaching 60–80% among pregnant women.

Vitamin D is a fat-soluble secosteroid which is essential not only for calcium–phosphate metabolism and musculoskeletal health but also for immune regulation and vascular function during pregnancy [4,5]. Vitamin D, beyond its well-established role in calcium homeostasis and bone metabolism, has been increasingly recognized for its pleiotropic effects on the immune system, endothelial function, and placental development [4,7]. Several studies have proposed a potential link between maternal VDD and adverse pregnancy outcomes, including gestational hypertension, preeclampsia, gestational diabetes, preterm birth, and impaired fetal growth [8]. VDD is increasingly recognized as a common issue even in sun-abundant regions such as Greece, with recent cross-sectional data reporting persistently low maternal and neonatal 25(OH)D levels at term despite supplementation [7]. Overall, although evidence regarding gestational hypertension remains inconsistent, maternal VDD is consistently reported as a prevalent issue. This is particularly evident in Mediterranean populations, such as Greece, where, despite abundant sunlight, lifestyle, cultural, and dietary factors [7] contribute to persistently low levels, a paradox that has intensified scientific interest in its impact during pregnancy.

To contextualize current evidence, recent studies have investigated the association between maternal vitamin D status and hypertensive disorders of pregnancy with variable results. Given the extensive but heterogeneous literature on this topic, the following section briefly summarizes the most representative findings from recent meta-analyses and large-scale studies to contextualize the present investigation. Previous evidence on the relationship between maternal vitamin D status and hypertensive disorders of pregnancy (HDP) is inconsistent. Umbrella reviews and meta-analyses have suggested possible associations with preeclampsia and other adverse outcomes [5,8], and some randomized trials indicate that supplementation may reduce risk [8]. However, several large cohort studies and systematic reviews reported null or inconclusive associations [9,10,11]. Overall, the literature remains heterogeneous, highlighting the need for context-specific investigations.

Similarly, a study by Lee et al. [12] involving 5169 pregnant women reported no significant association between maternal VDD and the risk of HDP. The study’s results imply that while VDD is common during pregnancy, it may not be a major contributor to the development of HDP.

Evidence from systematic reviews and meta-analyses has shown inconsistent findings. Some studies reported that low maternal 25(OH)D levels were associated with an increased risk of preeclampsia and gestational hypertension [13], whereas others, including large-scale randomized controlled trials, failed to demonstrate a clear causal relationship [13,14]. These discrepancies may be attributed to differences in study design, population characteristics, timing and dose of supplementation, and varying thresholds used to define VDD [15,16]. Several recent systematic reviews and meta-analyses support a protective role of vitamin D in hypertensive disorders of pregnancy. For instance, Moghib et al. [8] pooled 33 randomized controlled trials including more than 10,000 women and found a 45% reduction in preeclampsia risk with vitamin D supplementation (RR = 0.55, 95% CI: 0.43–0.71). Similarly, Fogacci et al. [17] reported a pooled odds ratio of 0.37 (95% CI: 0.26–0.52) in favor of supplementation. Observational data corroborate an increased risk: Akbari et al. [18], in their meta-analysis, found that VDD (<20 ng/mL) was associated with an elevated risk of preeclampsia, with a pooled OR of 1.54 (95% CI via random-effects model). Consistently, Hu et al. [19], in a systematic review and meta-analysis of 22 studies including more than 25,000 women, demonstrated that both vitamin D insufficiency and deficiency in early to mid-pregnancy were associated with significantly increased odds of preeclampsia, with ORs ranging from 1.35 to 1.58 depending on cutoffs.

In contrast, other evidence has failed to confirm this relationship. A randomized controlled trial in the Democratic Republic of Congo reported no significant difference in preeclampsia incidence between women receiving high-dose (4400 IU/day) versus low-dose (400 IU/day) vitamin D [20]. Similarly, a systematic review of observational studies yielded non-significant pooled estimates (OR = 0.78, 95% CI: 0.59–1.05), suggesting that the association may not be consistent across populations [21]. In addition, the Cochrane review by De-Regil et al. [13], which included randomized controlled trials, concluded that current evidence was insufficient to determine whether vitamin D supplementation reduces the risk of hypertensive disorders of pregnancy [13]. Taken together, these inconsistencies highlight the ongoing uncertainty and underscore the need for high-quality, adequately powered studies in diverse populations.

Taken together, current evidence indicates that vitamin D supplementation may exert a protective effect against preeclampsia in certain settings, yet the association with gestational hypertension appears weak or inconsistent. The heterogeneity of findings across studies likely reflects differences in design, population characteristics, timing and dosage of supplementation, and the thresholds applied to define deficiency. Considering the substantial prevalence of hypovitaminosis D during pregnancy, particularly in Mediterranean populations such as Greece, clarifying its role in hypertensive disorders of pregnancy remains a research priority. Despite the growing body of international literature, evidence from Mediterranean settings remains scarce and fragmented, highlighting the need for population-specific data. In Mediterranean countries such as Greece, the investigation of vitamin D status during pregnancy carries particular public health relevance [6,7,22]. Despite abundant year-round sunlight, lifestyle patterns including limited outdoor activity, urban living, use of sunscreen [23], and cultural clothing habits substantially restrict dermal synthesis of vitamin D [6,7]. Additionally, dietary intake is often insufficient due to low consumption of fortified foods and fish, and adherence to supplementation remains suboptimal [24]. These factors contribute to persistently high rates of vitamin D deficiency (VDD) among pregnant women in Greece [6,7,25] and other Mediterranean populations [26,27,28]. Understanding these region-specific determinants is therefore essential for clarifying the impact of maternal VDD on pregnancy outcomes within Mediterranean populations.

Consequently, studying the maternal–neonatal vitamin D relationship in the Greek population provides valuable insight into a region that is paradoxically sun-rich yet characterized by widespread hypovitaminosis D. This cross-sectional study therefore explores maternal and neonatal vitamin D (25-hydroxyvitamin D) status in an urban Greek population, focusing on its potential implications for hypertensive disorders of pregnancy, including gestational hypertension and preeclampsia.

## 2. Materials and Methods

### 2.1. Study Design and Participants

This cross-sectional study included 248 mother–infant dyads who delivered at Tzaneio General Hospital of Piraeus, Greece. Eligible participants were either of Greek origin or long-term residents (>10 years). Women with multiple pregnancies, known pre-existing hypertension, diabetes, or other chronic systemic conditions were excluded. Written informed consent was obtained from all participants, and the study was approved by the hospital’s ethics committee.

The final sample of 248 mother–infant dyads was determined based on feasibility within the study period and comparison with similar cross-sectional studies conducted in Mediterranean populations. Given the limited number of eligible deliveries meeting the inclusion criteria during the data collection window, this sample size was deemed sufficient to provide adequate statistical power for detecting moderate associations between maternal vitamin D status and hypertensive disorders of pregnancy. Comparable sample sizes have been previously reported in similar studies examining maternal–neonatal vitamin D correlations and pregnancy outcomes [6,7,8,18,25].

### 2.2. Data Collection

Demographic and clinical data were collected using a structured postpartum questionnaire, completed by mothers shortly after delivery, and supplemented by information from medical records. Variables included maternal age, parity, body mass index (BMI), smoking status, socioeconomic status, history of gestational complications (gestational hypertension, preeclampsia, placental abruption), and use of vitamin D supplementation during pregnancy.

### 2.3. Blood Sample Collection and Vitamin D Assessment

Maternal venous blood was obtained at delivery, and umbilical cord blood samples were collected immediately after birth. These measurements reflected vitamin D status at term rather than throughout gestation. Although trimester-specific variations were not assessed, the sample included births across both winter and summer seasons, allowing for comparison of seasonal fluctuations in maternal and neonatal 25(OH)D concentrations. Serum 25-hydroxyvitamin D [25(OH)D] concentrations were measured using [specify assay/method, e.g., chemiluminescent immunoassay] according to the manufacturer’s instructions. Maternal and neonatal vitamin D status were classified as deficient (<20 ng/mL), insufficient (20–29 ng/mL), or sufficient (≥30 ng/mL). For risk-stratified analyses, values < 30 ng/mL were considered low.

### 2.4. Definition of Hypertensive Disorders of Pregnancy

Gestational hypertension was defined as new-onset systolic blood pressure ≥ 140 mmHg or diastolic pressure ≥ 90 mmHg after 20 weeks of gestation without proteinuria. Preeclampsia was defined as gestational hypertension accompanied by proteinuria or other systemic manifestations according to international guidelines.

### 2.5. Statistical Analysis

Categorical variables were presented as counts and percentages, and continuous variables as means ± standard deviations. Associations between maternal vitamin D status (<20 vs. ≥20 ng/mL) and hypertensive disorders were assessed using chi-square tests. Multivariate logistic regression analyses were performed to evaluate the independent contribution of maternal vitamin D deficiency and other potential risk factors—including BMI, age, parity, smoking status, socioeconomic status, and supplementation use—on the likelihood of developing gestational hypertension or preeclampsia. Statistical significance was set at *p* < 0.05. All analyses were two-tailed with significance set at *p* < 0.05. Statistical analyses were performed using IBM SPSS Statistics version 26.0 (IBM Corp., Armonk, NY, USA) and R software (version 4.2).

## 3. Results

### 3.1. Descriptive Data

We analyzed 248 mother–infant dyads, all of Greek origin or long-term residents (>10 years). The mean maternal age was ~30 years, with the majority being nulliparous. Gestational hypertension was diagnosed in 29 women (11.7%) and preeclampsia in 15 women (6.0%). Eighty-three participants (33.5%) reported vitamin D supplementation during pregnancy. Detailed demographic and clinical characteristics are presented in Table 1.

Maternal serum 25(OH)D concentrations demonstrated marked seasonality, ranging from 16.96 ± 9.60 ng/mL in winter to 24.22 ± 12.57 ng/mL in summer. Neonatal cord blood levels were consistently lower (12.87 ± 8.20 vs. 16.37 ± 8.55 ng/mL). Across the cohort, 84% of mothers and 92% of neonates had levels < 30 ng/mL. Among supplemented women, mean levels were higher than in non-supplemented peers (26.9 ± 12.4 vs. 16.9 ± 9.6 ng/mL), though 73% of their neonates still remained <20 ng/mL. The distribution of vitamin D status across categories is shown in Table 2, while seasonal variation and prevalence of deficiency are summarized in Table 3.

### 3.2. Maternal–Neonatal Correlation

A strong positive correlation was observed between maternal and neonatal serum 25(OH)D concentrations (r = 0.80, *p* < 0.001).

### 3.3. Vitamin D and Hypertensive Disorders of Pregnancy

Women with gestational hypertension or preeclampsia were disproportionately represented among those with maternal 25(OH)D < 30 ng/mL. Mean maternal levels in preeclampsia were the lowest in the cohort (~15 ng/mL), with correspondingly low neonatal cord levels.

In univariate analyses, women with maternal vitamin D concentrations < 20 ng/mL had a significantly higher prevalence of gestational hypertension compared to those with ≥20 ng/mL (21.2% vs. 6.7%, *p* = 0.001; Table 4). A similar pattern was observed for preeclampsia (10.6% vs. 3.7%, *p* = 0.027; Table 5).

In multivariate logistic regression (Table 6), vitamin D deficiency remained an independent predictor of gestational hypertension (OR 2.15, 95% CI: 1.08–4.27, *p* = 0.028), alongside maternal BMI.

For preeclampsia, however, the association with vitamin D deficiency did not remain statistically significant after adjustment (OR 1.64, 95% CI: 0.72–3.74, *p* = 0.24). Higher maternal BMI was the only variable independently associated with preeclampsia risk (Table 7).

## 4. Discussion

This cross-sectional study aimed to investigate maternal and neonatal vitamin D status in a cohort of Greek women and to explore potential associations with hypertensive disorders of pregnancy. Our results demonstrate a high prevalence of maternal and neonatal vitamin D insufficiency, with more than four out of five women and over 90% of neonates presenting serum 25(OH)D levels below 30 ng/mL. Despite the fact that one-third of the mothers reported vitamin D supplementation, neonatal concentrations remained consistently low, highlighting the persistent gap between maternal and cord blood levels. This observation aligns with evidence from Moghib et al. [8] and Fogacci et al. [17], who suggested that fixed-dose supplementation may be insufficient to achieve optimal neonatal levels, highlighting the need for individualized dosing based on maternal baseline 25(OH)D and seasonal factors. These findings are consistent with our previous work [6], which also demonstrated that over 70% of pregnant women in Greece had serum concentrations below 30 ng/mL. Comparable prevalence rates have been reported in other Mediterranean populations and internationally, even in sun-rich regions [14,19]. Thus, the high frequency of maternal and neonatal deficiency observed in the present study aligns with global evidence indicating that adequate sun exposure does not necessarily translate into sufficient vitamin D status during pregnancy. This aligns with umbrella reviews including over 250,000 women, which demonstrated associations between maternal vitamin D deficiency and adverse outcomes such as preeclampsia, preterm birth, and small-for-gestational-age infants [5]. These reviews also highlighted substantial heterogeneity across populations, reinforcing the need for context-specific studies.

In line with previous research, our analysis showed that very low maternal vitamin D levels (<20 ng/mL) were significantly associated with gestational hypertension in univariate analysis, and this relationship persisted after adjustment for confounding factors such as body mass index (BMI), parity, smoking, and socioeconomic status. On the other hand, the association between VDD and preeclampsia, although evident in univariate comparisons, was attenuated and no longer statistically significant in the multivariate models, suggesting that additional maternal factors, particularly BMI, may play a more dominant role. These findings reinforce the multifactorial nature of hypertensive disorders in pregnancy, where nutritional, metabolic, and demographic variables interact in complex ways. Our findings regarding gestational hypertension are in line with those of Hu et al. [19] and Akbari et al. [18], who also reported an increased risk among women with severe VDD. Similarly, Moghib et al. [8] and Fogacci et al. [17] found that vitamin D supplementation was associated with a significantly lower incidence of preeclampsia. In contrast, a large U.S. cohort reported no overall association between maternal 25(OH)D and preeclampsia, although higher concentrations appeared protective against severe cases [9]. Similarly, a systematic review of 11 prospective cohorts found no consistent association between vitamin D status and hypertensive disorders of pregnancy (summary RR = 1.09, 95% CI: 0.84–1.41; I^2^ = 75%) [11]. A large Chinese cohort also reported null associations, but identified vitamin D receptor gene polymorphisms that may influence susceptibility to preeclampsia [10]. In addition, Lee et al. [12] emphasized maternal BMI as a more decisive predictor. Taken together, these mixed results mirror our own observations, where VDD remained independently associated with gestational hypertension but not with preeclampsia after adjustment. Thus, our study supports the notion that VDD may play a more prominent role in gestational hypertension than in preeclampsia, consistent with pooled analyses by Hu et al. [19] and Akbari et al. [18].

The observed strong correlation between maternal and neonatal 25(OH)D levels further underscores the dependency of neonatal vitamin D status on maternal supply. However, even among women who received supplementation, neonatal deficiency remained highly prevalent, suggesting suboptimal dosing, differences in absorption or metabolism, or delayed initiation of supplementation. Several interrelated factors likely contribute to this finding. First, many women likely initiated supplementation late in pregnancy, which may not allow sufficient time for maternal 25(OH)D concentrations to normalize and transfer to the fetus. Second, the fixed-dose regimens commonly prescribed in Greece (typically 400–800 IU/day) may be inadequate for women starting from low baseline levels, as supported by recent meta-analyses suggesting that doses above 1000–2000 IU/day are often required to achieve sufficiency [14,17,24]. In addition, adherence to supplementation can vary considerably due to nausea, forgetfulness, or lack of counselling, potentially contributing to suboptimal maternal and neonatal levels. Variations in timing, dosage, and compliance, therefore, represent key determinants of the persistent neonatal deficiency observed, underscoring the need for individualized supplementation strategies tailored to maternal baseline status and seasonal context. The strong maternal–neonatal correlation observed here is consistent with findings by Bi et al. [14] and Hu et al. [19], who similarly reported that neonatal vitamin D concentrations are largely dependent on maternal supply. This reinforces the critical role of optimizing maternal vitamin D status to ensure adequate neonatal levels.

The biological plausibility of these associations is supported by mechanistic studies suggesting that vitamin D influences placental function and immune regulation, mechanisms that may underlie its role in hypertensive disorders of pregnancy [8,15,16]. Liu et al. [15] demonstrated that vitamin D exerts immunomodulatory effects that may attenuate the inflammatory processes implicated in hypertensive disorders of pregnancy. Likewise, Gerovasili et al. [16] highlighted its role in maintaining endothelial function and vascular health. Such mechanisms provide a potential explanation for the associations observed, even though causal pathways remain to be clarified.

Our study has several strengths, including a well-defined cohort, the simultaneous assessment of maternal and cord blood samples, and the systematic evaluation of multiple confounders. Nevertheless, certain limitations must be acknowledged. The cross-sectional design precludes causal inference, and the associations observed cannot establish direct cause-and-effect relationships. The relatively modest sample size may also have limited the statistical power for less common outcomes, such as preeclampsia. Moreover, vitamin D intake was assessed only through self-reported supplementation without detailed dietary records, and we did not account for potential genetic polymorphisms affecting vitamin D metabolism.

Furthermore, other environmental and genetic determinants, such as dietary vitamin D intake, sunlight exposure, physical activity, and vitamin D receptor (VDR) polymorphisms, may also influence maternal 25(OH)D levels and susceptibility to hypertensive disorders of pregnancy. For instance, studies have shown that limited outdoor activity and low dietary intake of vitamin D–rich foods contribute to persistent deficiency even in sun-abundant regions [7,24,26]. Genetic polymorphisms in the VDR gene have been associated with altered vitamin D metabolism and increased preeclampsia risk [10]. Similarly, insufficient physical activity has been linked to both lower vitamin D concentrations and a higher incidence of gestational hypertension [29,30]. Although these factors were not directly assessed in the present study, their potential contribution cannot be excluded and warrants further investigation.

Additionally, although maternal physical activity was recorded in the postpartum questionnaire, it was collected in qualitative form (self-reported as increased or decreased) and therefore not included in the multivariable analysis. Nonetheless, regular physical activity has been associated with reduced risk of gestational hypertension and preeclampsia in previous studies [29,30,31], and future studies should incorporate standardized assessment tools to better account for this confounder.

Moreover, sleep quality was not assessed in the present study, representing another potential source of residual confounding, as poor sleep has also been linked to an increased risk of hypertensive disorders in pregnancy [32].

Furthermore, vitamin D concentrations were measured only at the time of delivery, reflecting maternal and neonatal status at term rather than throughout pregnancy. Although trimester-specific variations could not be captured, the inclusion of both winter and summer deliveries allowed for the assessment of seasonal fluctuations in 25(OH)D levels, partially addressing temporal variation.

Finally, the study’s single-centre design and ethnically homogeneous sample may limit the generalisability of findings to other Mediterranean or multi-ethnic populations. Although Greece shares environmental and cultural similarities with neighbouring regions, variations in sunlight exposure, dietary habits, supplementation practices, and genetic determinants of vitamin D metabolism across Mediterranean countries could influence maternal and neonatal 25(OH)D levels [26,27,28]. Therefore, future multi-centre studies across different Mediterranean settings are warranted to confirm the external validity of these findings.

Despite these limitations, the present findings contribute to the growing body of evidence linking VDD with adverse pregnancy outcomes. Given the particularly high prevalence of hypovitaminosis D in our population, routine monitoring and evidence-based supplementation strategies may warrant consideration. Specifically, studies examining individualized supplementation strategies, gene–nutrient interactions, and the influence of seasonal and lifestyle factors will help clarify causality and optimize maternal–fetal outcomes. From a clinical perspective, the development of individualized supplementation strategies should consider baseline maternal 25(OH)D levels, gestational timing, and seasonality. Current evidence suggests that maintaining maternal serum 25(OH)D concentrations above 30 ng/mL (75 nmol/L) is associated with lower risk of preeclampsia and improved neonatal status [8,17,24]. Several systematic reviews and randomized controlled trials indicate that daily doses of 1000–2000 IU, or equivalent intermittent regimens, are generally safe and effective for achieving sufficiency during pregnancy [13,14,17]. Nonetheless, dose requirements may vary depending on baseline deficiency, BMI, and adherence, supporting the need for individualized counselling and regular biochemical monitoring throughout gestation. Future prospective studies and randomized controlled trials are necessary to clarify the causal pathways and to determine optimal maternal vitamin D targets for the prevention of hypertensive disorders of pregnancy.

## 5. Conclusions

In this cross-sectional study, we found that VDD was highly prevalent among Greek pregnant women and their neonates. Very low maternal 25(OH)D concentrations (<20 ng/mL) were independently associated with gestational hypertension, while the association with preeclampsia was less robust after adjustment for confounders, underscoring the multifactorial nature of these complications.

These findings emphasize the importance of systematically addressing vitamin D status during pregnancy as part of comprehensive prenatal care. Evidence from previous research suggests potential benefits of adequate vitamin D levels for maternal outcomes, while our results highlight the persistent challenge of achieving optimal neonatal concentrations despite supplementation. Routine monitoring and tailored supplementation strategies, adapted to maternal baseline status and seasonal context, may contribute to improved maternal and neonatal health outcomes.

Although these findings highlight important associations between maternal and neonatal vitamin D status and hypertensive disorders of pregnancy, the cross-sectional design precludes causal inference, and longitudinal or interventional studies are required to confirm these relationships.

## Figures and Tables

**Table 1:** Demographic and clinical characteristics of the study population (n = 248).

Characteristic	n (%) or Mean ± SD
Maternal age (years)	30.8 ± 5.1
<25	42 (16.9)
25–34	142 (57.3)
≥35	64 (25.8)
BMI (kg/m^2^)	26.4 ± 4.8
<25 (Normal)	110 (44.4)
25–29.9 (Overweight)	83 (33.5)
≥30 (Obese)	55 (22.2)
Parity	
Nulliparous	138 (55.6)
Multiparous	110 (44.4)
Smoking during pregnancy	
Yes	61 (24.6)
No	187 (75.4)
Socioeconomic status	
Low	72 (29.0)
Middle	126 (50.8)
High	50 (20.2)
Vitamin D supplementation	83 (33.5)
Gestational hypertension	29 (11.7)
Preeclampsia	15 (6.0)

Data are presented as mean ± standard deviation (SD) for continuous variables and n (%) for categorical variables.

**Table 2:** Maternal and neonatal vitamin D status (n = 248).

Category	Maternal Serum 25(OH)D n (%)	Neonatal Cord 25(OH)D n (%)
<20 ng/mL	116 (46.8)	142 (57.3)
20–29 ng/mL	93 (37.5)	86 (34.7)
≥30 ng/mL	39 (15.7)	20 (8.1)
Mean ± SD (ng/mL)	20.4 ± 11.2	14.5 ± 8.6

Data are presented as n (%) for categorical vitamin D categories and as mean ± SD for continuous 25(OH)D values.

**Table 3:** Maternal and neonatal 25(OH)D concentrations and prevalence of deficiency (<30 ng/mL).

Variable	n (%) or Mean ± SD
Maternal age (years)	30.8 ± 5.1
Nulliparous	138 (55.6%)
Supplement use	83 (33.5%)
Gestational hypertension	29 (11.7%)
Preeclampsia	15 (6.0%)
Maternal 25(OH)D (ng/mL)	20.4 ± 11.2
– Winter	16.96 ± 9.60
– Summer	24.22 ± 12.57
Neonatal 25(OH)D (ng/mL)	14.5 ± 8.6
– Winter	12.87 ± 8.20
– Summer	16.37 ± 8.55
Maternal < 30 ng/mL	209 (84.3%)
Neonatal < 30 ng/mL	228 (91.9%)

Values are presented as mean ± SD for continuous variables and n (%) for categorical variables. Seasonal comparisons were assessed using an independent-samples *t*-test for normally distributed variables (see Section 2).

**Table 4:** Association between maternal vitamin D status (<20 vs. ≥20 ng/mL) and gestational hypertension.

Maternal 25(OH)D	Gestational Hypertension n (%)	No Gestational Hypertension n (%)	Total n (%)
<20 ng/mL (n = 85)	18 (21.2%)	67 (78.8%)	85 (34.3%)
≥20 ng/mL (n = 163)	11 (6.7%)	152 (93.3%)	163 (65.7%)
Total (n = 248)	29 (11.7%)	219 (88.3%)	248 (100%)

Relative frequencies are expressed as percentages (%) for categorical variables. Statistical analysis was performed using the Chi-square test. *p* < 0.05 denotes statistical significance.

**Table 5:** Association between maternal vitamin D status (<20 vs. ≥20 ng/mL) and preeclampsia.

Maternal 25(OH)D	Preeclampsia n (%)	No Preeclampsia n (%)	Total n (%)
<20 ng/mL (n = 85)	9 (10.6%)	76 (89.4%)	85 (34.3%)
≥20 ng/mL (n = 163)	6 (3.7%)	157 (96.3%)	163 (65.7%)
Total (n = 248)	15 (6.0%)	233 (94.0%)	248 (100%)

Relative frequencies are expressed as percentages (%) for categorical variables. Statistical analysis was performed using the Chi-square test. *p* < 0.05 denotes statistical significance.

**Table 6:** Multivariate logistic regression for predictors of gestational hypertension (n = 248).

Variable	OR	95% CI	*p*-Value
Vitamin D < 30 ng/mL	2.15	1.08–4.27	0.028 *
Supplement use (yes)	0.76	0.39–1.48	0.42
BMI (per 1 kg/m^2^)	1.09	1.02–1.16	0.009 **
Maternal age (per year)	1.03	0.96–1.10	0.41
Smoking (yes)	1.42	0.69–2.94	0.34
Nulliparous (vs. multiparous)	1.21	0.62–2.38	0.57
Low SES (vs. middle/high)	1.67	0.81–3.45	0.16

Results are presented as adjusted odds ratios (OR) with 95% confidence intervals (CI). Significant predictors are indicated by * *p* < 0.05 and ** *p* < 0.01. Model covariates: vitamin D status (<30 ng/mL), supplementation (yes/no), BMI (per 1 kg/m^2^), maternal age (per year), smoking (yes/no), parity (nulliparous vs. multiparous), and socioeconomic status (low vs. middle/high).

**Table 7:** Multivariate logistic regression for predictors of preeclampsia (n = 248).

Variable	OR	95% CI	*p*-Value
Vitamin D < 30 ng/mL	1.64	0.72–3.74	0.24
Supplement use (yes)	0.71	0.29–1.75	0.46
BMI (per 1 kg/m^2^)	1.12	1.04–1.21	0.004
Maternal age (per year)	1.05	0.96–1.15	0.27
Smoking (yes)	1.31	0.51–3.37	0.57
Nulliparous (vs. multiparous)	1.84	0.79–4.27	0.15
Low SES (vs. middle/high)	1.93	0.82–4.57	0.13

Results are presented as adjusted odds ratios (OR) with 95% confidence intervals (CI). Significant predictors are indicated by *p* < 0.05 and *p* < 0.01. Model covariates: vitamin D status (<30 ng/mL), supplementation (yes/no), BMI (per 1 kg/m^2^), maternal age (per year), smoking (yes/no), parity (nulliparous vs. multiparous), and socioeconomic status (low vs. middle/high).

## Data Availability

The raw data supporting the conclusions of this article will be made available by the authors on request.
